# Role of cholesterol and sphingolipids in brain development and neurological diseases

**DOI:** 10.1186/s12944-019-0965-z

**Published:** 2019-01-25

**Authors:** Ghulam Hussain, Jing Wang, Azhar Rasul, Haseeb Anwar, Ali Imran, Muhammad Qasim, Shamaila Zafar, Syed Kashif Shahid Kamran, Aroona Razzaq, Nimra Aziz, Waseem Ahmad, Asghar Shabbir, Javed Iqbal, Shahid Mahmood Baig, Tao Sun

**Affiliations:** 1 Department of Physiology, Faculty of Life Sciences, Government College University, Faisalabad, Pakistan; 2 Center for Precision Medicine, School of Medicine and School of Biomedical Sciences, Huaqiao University, Xiamen, 361021 Fujian Province China; 3 Department of Zoology, Faculty of Life Sciences, Government College University, Faisalabad, Pakistan; 4 Institute of Home and Food Sciences, Government College University, Faisalabad, Pakistan; 5 Department of Bioinformatics and Biotechnology, Government College University, Faisalabad, Pakistan; 6 Department of Biosciences, COMSATS Institute of Information Technology, Islamabad, Pakistan; 7 Department of Neurology, Allied Hospital, Faisalabad, Pakistan; 8 Human Molecular Genetics Laboratory, Health Biotechnology Division, National Institute for Biotechnology and Genetic Engineering (NIBGE), PIEAS, Faisalabad, Pakistan

**Keywords:** Cholesterol, Sphingolipids, Development, Neurological diseases, Nervous system

## Abstract

Brain is a vital organ of the human body which performs very important functions such as analysis, processing, coordination, and execution of electrical signals. For this purpose, it depends on a complex network of nerves which are ensheathed in lipids tailored myelin; an abundant source of lipids in the body. The nervous system is enriched with important classes of lipids; sphingolipids and cholesterol which compose the major portion of the brain particularly in the form of myelin. Both cholesterol and sphingolipids are embedded in the microdomains of membrane rafts and are functional units of the neuronal cell membrane. These molecules serve as the signaling molecules; hold important roles in the neuronal differentiation, synaptogenesis, and many others. Thus, their adequate provision and active metabolism are of crucial importance in the maintenance of physiological functions of brain and body of an individual. In the present review, we have highlighted the physiological roles of cholesterol and sphingolipids in the development of the nervous system as well as the association of their altered metabolism to neurological and neurodegenerative diseases.

## Introduction

Brain is an eminent organ of the human body; it comprises of more than 100 billion nerve cells which communicate by well-known structures called synapses. It serves as the primary center for initiation, coordination, interpretation, and integration of most of the nerve messages [1]. It controls many unconscious functions of the body such as respiratory process and heart rate. It also synchronizes most of the voluntary activities [2]. Interestingly, this complex and important system of the human body is highly enriched in lipid contents and is next to the adipose tissue [3–5]. Lipids account for 50–60% of its dry weight [6] and a substantial amount of lipids in the nervous system is present in the myelin sheath, a particular form of membrane acquiring maximum lipids among entire biological membranes [7]. Lipids found in the brain are grouped as sphingolipids, glycerophospholipids, and cholesterol and are considered to be present in almost equal ratios [8, 9]. These lipids are involved in developmental, maintenance and many other cellular processes of the brain. The lipids act as signaling molecules, source of energy, for contributing to synaptogenesis, neurogenesis, impulse conduction and many others [1, 7]. Furthermore, lysophospholipids, endocannabinoids, and sphingolipids (SP) have also been reported to be involved in cellular signaling, including regulation of numerous ion pumps, channels, and transporters [10, 11]. All vital events responsible for the development and maintenance of functional activities of the nervous system depend on the unique lipid contents found in the different membrane regions (lipid rafts) of neuronal cells. Any change in lipids metabolism results in altered lipid composition of intracellular membrane compartments which is a common biomarker in many neuronal disorders [3, 4]. Lipid rafts found in the neuronal cell membrane containing cholesterol and sphingolipids (particularly glycosphingolipids) [9, 12, 13]. Moreover, it has long been known that myelin structure and brain homeostasis rely on specific lipid-protein interactions and on specific cell-to-cell signaling [13]. Most of the CNS disorders and injuries are linked with impaired lipid metabolism like Alzheimer’s disease (AD), Parkinson’s disease (PD), Huntington’s disease (HD), schizophrenia, epilepsy, and bipolar disorders in which progressive degeneration of neurons occurs [3, 14].

## Cholesterol

Cholesterol, a vital constituent for normal functioning of the nervous system, plays an important role both during the developmental stage and in adult life [9]. Brain contains about 25% of the whole body’s cholesterol and is considered as a cholesterol-rich organ [15]. Cholesterol is the most important component and fundamental functional unit of the mammalian cell membrane [16]. Most of the body cholesterol resides in brain in the form of myelin [17] which contains almost 80% of cholesterol found in adult brain [13]. Therefore, it is a key constituent of myelin in CNS and PNS which is synthesized by oligodendrocytes and Schwan cells respectively [13]. In human and other rodents, cholesterol is synthesized actively in CNS during first few weeks of post birth, and at this neonatal stage, any interruption in its synthesis and provision can lead to the development of neurodegenerative disorders (NDDs) [18]. From this, it can be surmised that cholesterol is required for cellular processes e.g., glial cell proliferation, neurite outgrowth, microtubules stability, synaptogenesis and myelination [19]. A vast series of studies suggests that cholesterol availability in oligodendrocytes functions as a limiting factor in brain maturation, myelination and neurotransmission [20]. Cholesterol may be synthesized endogenously or it can be exogenously supplied by endocytosis of plasma lipoproteins; for example low density lipoproteins (LDLs) mediated by particular receptors [21]. The rate of cholesterol synthesis depends on the ongoing myelination process and the excessive cholesterol is exported in the form of 24-hydroxycholesterol for maintaining the homeostasis of cholesterol [22, 23]. The neurons can synthesize only a minute quantity of cholesterol themselves and mostly rely on cholesterol-containing lipoproteins secreted by astrocytes [24].

Cholesterol metabolism in CNS is different from that in PNS. The blood brain barrier (BBB) hinders the passage of plasma lipoproteins to CNS, therefore, cholesterol requirement of CNS is met with locally synthesized cholesterol [25]. In CNS, the transport of cholesterol is carried by special lipoproteins such as Apolipoprotein-E (Apo-E) that are secreted by astrocytes [26–28]. The cholesterol-Apo-E complex accelerates axonal extension when applied to distal end but not to the cell body of neurons [29, 30].

**Fig. 1:**
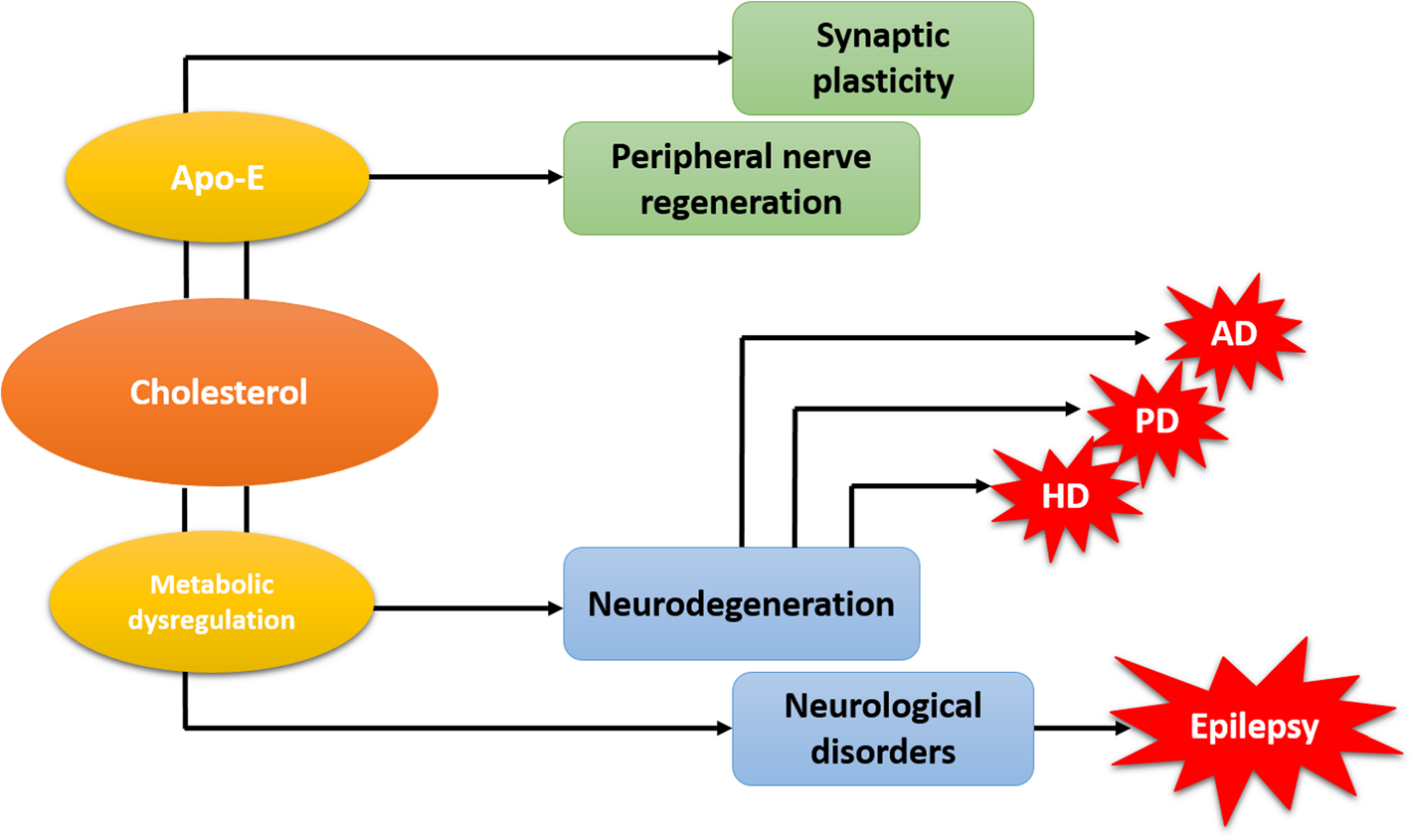
Role of cholesterol in brain health and disorders

The regeneration is the healing or complete replacement of damaged neural cell. It appears a basic requirement for maintaining a normal physiology at post injury stage, but unfortunately, this ability is limited in higher organisms including human [31]. The neurons in CNS lose their ability to regenerate early in development and underlying mechanisms of this loss are poorly understood till date [32]. In this regard, a study demonstrated that this limitation of neural regeneration is associated with the expression of Nogo-A and NgR1 receptors following an injury to CNS [33]. The delay in axons regeneration is a major obstacle for functional recovery after such injuries [34] that can result a long lasting disability [14]. Cholesterol regulates a large number of pathways that play key roles in brain health. Similarly, it also plays an important role in nerve regeneration. Although, cholesterol and its metabolites impact nerve regeneration positively, but it is also noteworthy to state that metabolic dysregulation of cholesterol is considered as a causative factor of several major brain disorders as described in Fig. 1.

### Cholesterol in synaptogenesis

Synaptogenesis is the process of synapse formation required for the functional development of brain. Cholesterol dependent synaptogenesis and cholesterol availability are limiting factors in the development of synapses in brain. Glial cells provide plenty of cholesterol in the form of cholesterol-Apo-E complex for massive synaptogenesis [28, 35]. The glia-derived cholesterol promotes the development of synapses in highly purified retinal ganglion cells (RGCs). This study also shows that cholesterol enhances the presynaptic differentiation. It is vital for a continuous synaptogenesis and important for the stability of neurotransmitters. These findings clearly demonstrate that cholesterol plays a key role in neuronal differentiation and plasticity [19]. An adequate availability of cholesterol is necessary for normal neuronal function and morphology. Neuronal functions are impaired not only by its deficiency but also due to excessive level [36, 37]. Cholesterol level in the brain is strictly monitored by various factors. Brain-Derived Neurotrophic Factor (BDNF) mediated cholesterol biosynthesis in CNS serves as a repository for the development of synaptic vesicles [38]. The shreds of evidence show that pre and postsynaptic areas are rich in cholesterol that maintains and organizes the synaptic proteins. This influences the neurotransmission and synaptic plasticity which subsequently, facilitates normal development of cognitive abilities [39].

### Cholesterol in peripheral nerve injury

Peripheral nerve injury (PNI) can have a potentially devastating impact on a patient’s quality of life, resulting in severe disability with substantial social and personal cost [40]. The requirement of cholesterol is increased in the case of nerve regeneration as it is an important modulator of axon regeneration following nerve injury [41]. Cholesterol plays a crucial role in the regeneration of nerve after injuries both in CNS and PNS. Local availability of cholesterol at nerve damage is necessary for nerve regeneration [42]. It is met by the increased supply of cholesterol in the form of lipoproteins from macrophages that recycle the cholesterol of degenerating neurons. It has been found that injured nerve responds to the elevated supply of exogenously provided cholesterol. The cholesterol contents of plasma membrane play a key role in synaptophysin-synaptobrevin complex formation that regulates the synaptic vesicle recycling for neurotransmitters release [43]. In this context, the regulation of lipids particularly cholesterol may provide a relief against neurodegenerative diseases or other disorders pertaining to nerve problems. Interestingly, some studies have also demonstrated that partial recovery is possible following a nerve damage.

The cholesterol-rich transporter lipoprotein Apo-E has been reported to accumulate at the site of injury after nerve crush [44]. Simply increasing the availability of cholesterol contributes to regeneration and remyelination and it strengthens the idea that regulating cholesterol availability after injury may help to recover injured PNS [45]. The role of Apo-E in maturation and regeneration of sciatic nerve is confirmed by many studies. After nerve injury, the cholesterol of dying axon is taken up by Schwann cells and resident macrophages. This can be reutilized for regeneration of axon and is transported mainly by Apo-E. The Apo-E is synthesized by macrophage and accumulated at the site of regenerating axon and it increases following an injury. Probably, efficient lipid transportation plays a key role in regenerating nerve because the LDL receptors are found clustered at the tip of growing axon. The expression of Apo-E increases with the injury and declines when regeneration ends [46]. However, another study indicates the involvement of unknown receptors other than low-density lipoproteins receptors (LDLR) which were thought to be primarily involved in uptake of cholesterol by Schwann cells for the purpose of nerve regeneration [47].

In the event of a crush injury and abnormal cholesterol transport; the peripheral nerve shows delayed axonal regeneration, but remyelination is not affected by cholesterol unavailability [48]. This shows that de novo synthesis of cholesterol by Schwann cells is enough for remyelination, but axonal growth is affected by cholesterol unavailability. This lays the foundation of the idea that controlling cholesterol transportation or improving its availability in neurodegenerative diseases (NDDS) can potentially facilitate the protection against the disease or even leads to delayed onset of disease.

### Cholesterol in neurodegenerative diseases

Although cholesterol has been shown to be positively associated with the physiological functions of the brain, however, any alteration in its metabolism leads to the onset of various brain ailments as discussed below:

#### Alzheimer’s disease

One of the major NDDs is Alzheimer’s disease (AD), which is characterized by the β-amyloid (Aβ) peptides aggregation and senile plaques [1, 49]. The plaques formation in AD results in damaged neurites and synapses. Importantly, Aβ is formed by the successive cleavage of Amyloid precursor proteins (APP) and the β-secretase (beta-site amyloid precursor protein cleaving enzyme, BACE) which is mediated by gamma secretase enzyme [50]. It is an established fact that abnormal cholesterol metabolism plays a crucial role in the development of AD. The dysregulation of cytosolic calcium level occurs in the astrocytes in case of AD which leads to the neuronal death. High level of membrane cholesterol leads to the incorporation of Aβ into the membranes and also enhances the cytosolic calcium that causes neuronal cell death [51, 52]. Apo-E is the main carrier, involved in the transportation of cholesterol and has three isoforms known as ε2, ε3, and ε4. A person who carries the ε4 allele in his genome is at high risk of developing AD. This lipoprotein binds to the numerous receptors on cell surface for the delivery of cholesterol and also to the Aβ peptide which initiates the events of toxicity and leads to the neurodegeneration and synaptic dysfunction in AD [53]. It also influences the cholesterol metabolism and leads to the formation of oxidation products of cholesterol (oxysterols) [54]. Studies reveal that diet containing high cholesterol enhances the level of Aβ peptides. Concentration of serum cholesterol rises approximately 10% higher in AD patients than that of healthy individuals [55]. Moreover, aggregation of cholesterol in the endosomal-lysosomal system leads to the altered APP processing and generation of Aβ peptides and also triggers the degeneration [56]. Additionally, independent of Apo-Eε4, the increased concentration of LDL and attenuated concentration of HDL are linked with the Aβ- indices [57, 58]. Hence, it can be stated that low LDL-C diet may prove to be significant in modulating the symptoms of AD.

#### Parkinson’s disease

**Fig. 2:**
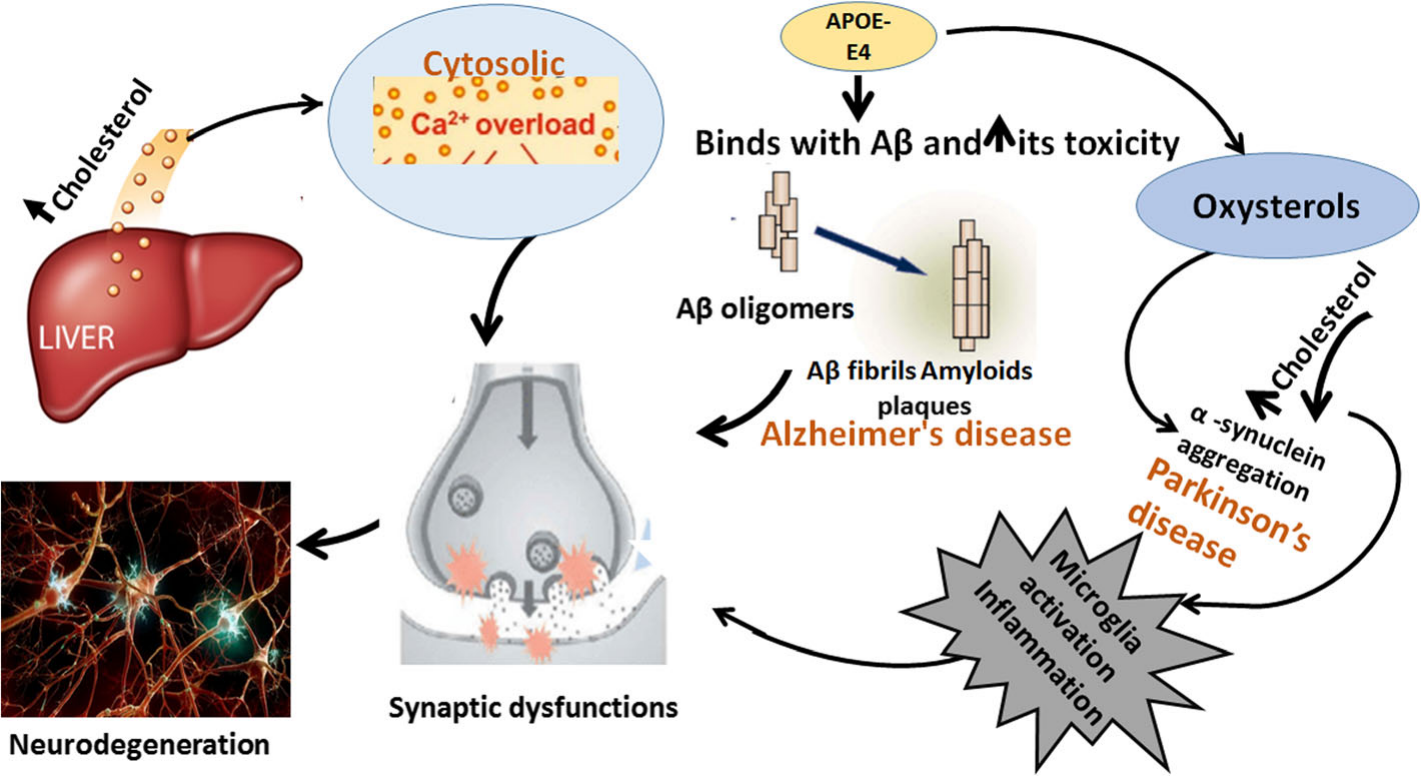
Role of cholesterol in Alzheimer’s and Parkinson’s disease

Parkinson’s disease (PD) is the second most prevalent NDDs after AD. Its pathology involves the loss of dopaminergic neurons in SN (substantia nigra) and the accumulation of α-synuclein and formation of Lewy bodies as well [59]. It was reported that high level of lipid prompts the accumulation of α-synuclein, the main constituent of Lewy bodies, by stimulating nucleation [60]. Most recent data demonstrate that high level of cholesterol and its oxidized product (oxysterol) play a crucial role in the development of PD by α-synuclein aggregation. It also causes inflammation, increase oxidative stress, and leads to the death of dopaminergic neurons [61, 62]. The previously said fact is supported by the recent evidences that higher cholesterol and oxysterols initiate the pathological pathways of α-synuclein aggregation adding in PD severity. Oxysterols initiate several pathological pathways like cell death followed by inflammation and oxidation ultimately α-synuclein aggregation. From all of these data, it can be concluded that higher cholesterol and oxysterols are taken as the major contributor of PD pathogenesis and also serve as potential biomarkers [61, 63, 64] Fig. 2.

#### Huntington’s disease

Huntington’s disease (HD) belongs to the family of NDDs, is caused by a mutation of autosomal dominant allele and by the abnormal extension of CAG repeat in Htt (huntingtin) gene. It is characterized by the degeneration of neurons of cortex and striatum regions [65]. The cholesterol metabolism is impaired in HD and is proportional to the length of CAG repeat. Its level is reduced at the advanced stage of HD [66, 67]. Recently, a research was conducted to measure the synthetic precursors, its metabolites and oxidation products of cholesterol in the five areas of the human postmortem brain of HD. Hence, it can be concluded that human brain with HD has considerably reduced cholesterol metabolism.

#### Neurological and psychiatric disorders

A subtype of glutamate receptors named N-methyl-D-aspartate receptors (NMDARs), which mediates excitatory neuronal transmission, plays a critical role in brain functions [68]. Neurological and psychiatric disorders such as stroke, schizophrenia and certain forms of autism can be attributed to dysfunctional NMDARs [69, 70]. The 24S-hydroxycholesterol, a brain cholesterol metabolite, is a positive modulator of NMDARs when administered exogenously [71, 72]. A study demonstrates the modulatory effect of endogenous 24S-hydroxycholesterol on NMDARs activity and it modulates NMDAR-mediated functions. It can be a potential therapeutic target for the treatment of neuropsychiatric disorders [73]. It has been reported that LDL-C deficiency caused by a mutation in 3-hydroxy-3-methylglutaryl-CoA reductase (involved in LDL-C) and proprotein convertase subtilisin kexin 9 genes (involved in LDL-C) is linked with the enhanced risk of both neurological diseases and NDDs. A study reports that reduced LDL-C level is linked with the enhanced risk of epilepsy [74]. However, no recent data is available on this aspect.

### Sphingolipids

Sphingolipids (SP) are the composite molecules essentially found in all eukaryotes and a few of the viruses and prokaryotes. This class of lipids is derived from cell membrane lipids named as sphingomyelin (SM) [75]. Hydrophobic ceramide chain is a common molecule in their backbone structure. Synthesis of SP requires Palmitoyl-CoA and l-serine. Although, l-serine is not classified as an essential amino acid, but its external supply is vital for the synthesis of phosphatidylserine (PS) and sphingolipids in the specific types of neurons [76]. There are several hundred different types of SP, some of them play important roles in a variety of physiological processes. For example, ceramide, sphingosine (Sph), Sph-1-phosphate (S1P) and Cer-1-phosphate (C1P) function as bioactive molecules in different cellular processes e.g., regulation of signal transduction pathways, protein sorting to the mediation of cell-to-cell interactions and recognition [77, 78]. They contribute structurally to membrane lipids bilayer and lipid rafts which play a regulatory role in cellular functions [79]. SP are ubiquitous and play an essential role for the proper brain function and development. They are not only the vital structural components of plasma membranes but are also renowned as important regulators of various cellular events because of their capability to make microdomains in the plasma membrane. They are involved in neuronal differentiation and synaptic transmission in neuronal-glial connections and are also associated with myelin stability. Thus, any perturbation in SP metabolism can lead to the rearrangements of plasma membrane and development of various neurological diseases [79]. Pathological changes in the normal metabolism of SP and their homeostasis are the common factors leading to progression of schizophrenia and metabolic syndrome. They have important structural and functional role in cell cycle and inflammatory processes which are supposed to be part of pathophysiological processes involved in the development of such diseases [80].

### Sphingolipids in synaptogenesis

**Fig. 3:**
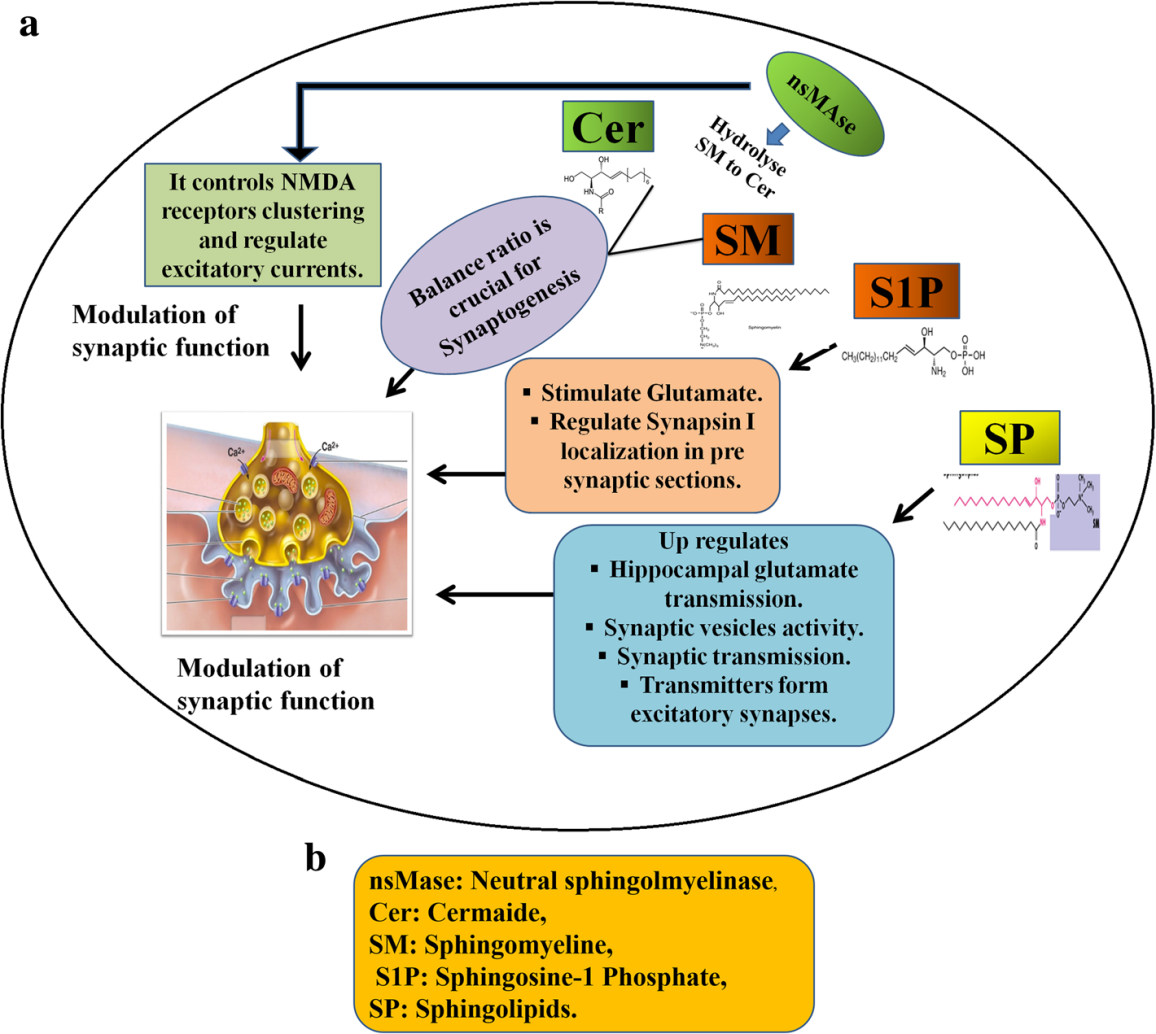
Role of Sphingolipids on Synaptic function and formation

The process of synaptogenesis usually occurs throughout the lifespan of a healthy individual but it is critical during early development of the brain. In this regard, a well-known class of lipids named as SP plays an important role. Synaptogenesis and synaptic plasticity involve various phenomenon such as recurrent presynaptic stimulation and long-term potentiation [81]. Importantly, neuronal plasticity serves as synapse efficacy modulation and is governed by the composition of synapse structure. SP are intimately involved in the neuronal membranes organization. Thus, not surprisingly, alterations in their pathways have been linked with the neuronal plasticity disturbances. Neutral sphingomyelinase-2 (nSMase) is present in the hippocampus and hydrolyzes the sphingomyelin (SM) to ceramide and modulates the postsynaptic function [82]. It regulates the postsynaptic excitatory currents by controlling NMDA receptors clustering and membrane insertion [83]. Some evidences suggest that nSMase deficient mice exhibit impaired episodic-like and spatial compromised plasticity [84]. It is vital to keep the balance between ceramide and SM for the maintenance of normal status [81]. Moreover, recent work suggests that SP also stimulates the release of transmitters to the terminals of excitatory synapses. They up-regulate the hippocampal glutamate transmission rate and also stimulate the release of glutamate from synaptosomes [85]. Interestingly, sphingosine-1-Phosphate (S1P) regulates the localization of synapsin-I in pre-synaptic compartments and hence it has the greatest impact on the modulation of presynaptic functions. S1P and SP stimulate the release of glutamate from the synaptosomes along with an incredible increase in the hippocampal glutamate transmission [85–87]. All these findings have helped to define the role of SP in the regulation of synaptic activity. Now, it can be concluded that its presence at presynaptic sites influences the activity of synaptic vesicle and stimulates the synaptic transmission. The following figure illustrates the modulation of synaptic functions by SP Fig. 3 a and b.

### Sphingolipids in neurogenesis

**Fig. 4:**
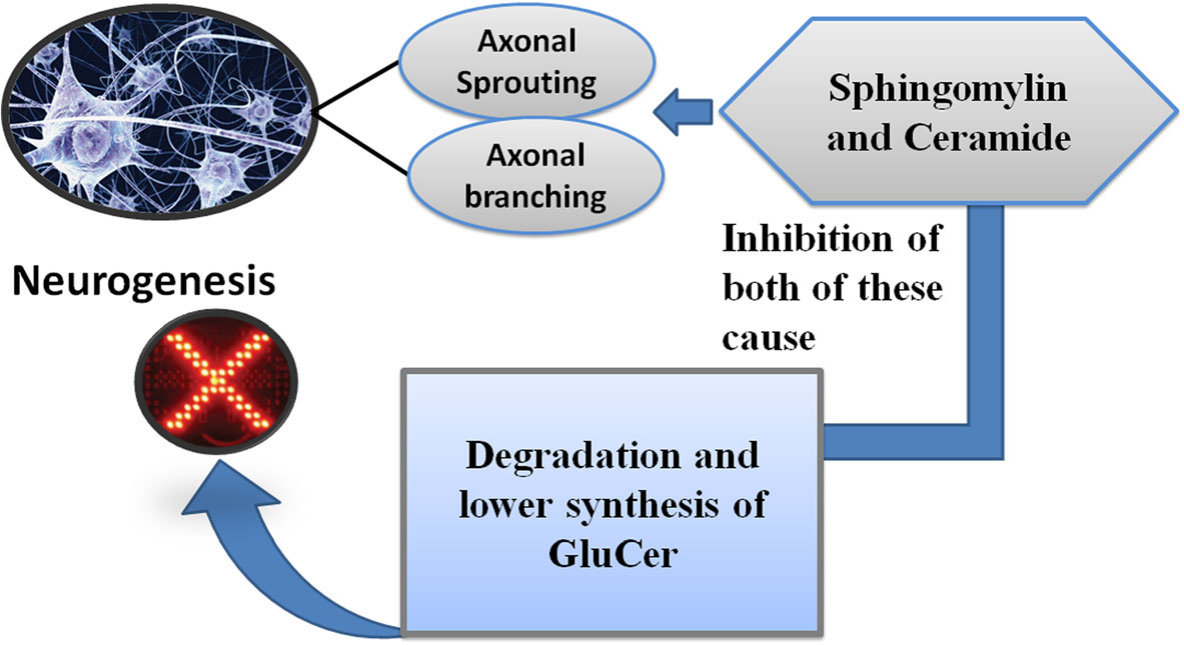
Role of sphingolipids in neurogenesis

Mammalian brain includes resident neural stem cells (NSCs) which facilitates the development and functional maturity of neurons in embryonic expansion and this process continues throughout adult life [88]. Axonal projection and outgrowth from the neuronal soma are important features in the formation of the neural network and plasticity. In this context, the role of SP is of considerable pertinence as they are an important constituent of the brain. Namely, SM and ceramide are involved in the so-called process of neurogenesis. Importantly, in hippocampal neuronal culture, SM and ceramide depletion condenses the outgrowth of axon [79]. However, the underlying mechanism may involve the combined inhibition of the GluCer synthesis. This feature favors the decreased axonal outgrowth along with the neuronal branching [89]. Moreover, in this regard, one of the hypothesis suggests that the inhibition of ceramide synthesis leads to the ceramide precursor accumulation, which possibly restricts the axonal growth [79]. Role of SP in neurogenesis as mentioned above is illustrated in Fig. 4.

### Sphingolipids and neurodegenerative diseases

It is important to safeguard the proper metabolism, composition, and integrity of sphingolipids for the maintenance of physiological functions the brain. Not surprisingly, defects in the metabolism of SP have been associated with numerous neurological diseases like PD, AD, and HD.

#### Parkinson’s disease

Generally, it is speculated that exact cause of PD is still unknown, but the neurodegeneration followed by the fibrillation and accumulation of α-synuclein in neurons is suggested to be the main contributing factor [49, 90]. It has been postulated that vesicle trafficking, lysosomes/autophagy, and mitochondrial dysfunction are the key factors which contribute to the accumulation of α-synuclein [91]. The enzyme which degrades the glycolipid glucosylceramide (GlcCer) is encoded by a gene known as glucocerebrosidase-1 (GBA1) which is one of the hallmarks in the pathology of PD. Hence, GlcCer facilitates the toxic alteration of α-synuclein. Furthermore, the GBA1 mutation may act as a genetic factor and may also enhance overall risk of PD by 5 to 6 folds [92]. The lysosomal enzyme which is encoded by GBA1 is glucocerebrosidase (GCase), predominantly expressed in several types of cells. Within the lysosome, it converts GluCer into glucose and ceramide. Moreover, the mutations in GBA1 are heterozygous in patients with GBA1-associated PD [93]. Recently, it has been shown that reduced activity of GCase is linked with the aggregation of α-synuclein. The insufficient level of GCase leads to the attenuation of ceramide level and mutation of intra-cellular constrains of Rab8a (minor GTPase) which is concerned with the secretory autophagy. All these factors impair the α-synuclein secretion and ultimately led to its intracellular aggregation [94]. Moreover, the diminished activity of GCase also impacts the activity of protein phosphate 2A (PP2A) via ubiquitous dysfunction of lysosomes, thereby increasing the accumulation of α-synuclein [95]. So, in the nutshell, it can be summarized that reduced generation of ceramide is due to mutation in GCase and PP2A contributes to the accumulation of α-synuclein, particularly due to the impairment in secretory autophagy.

#### Alzheimer’s disease

**Fig. 5:**
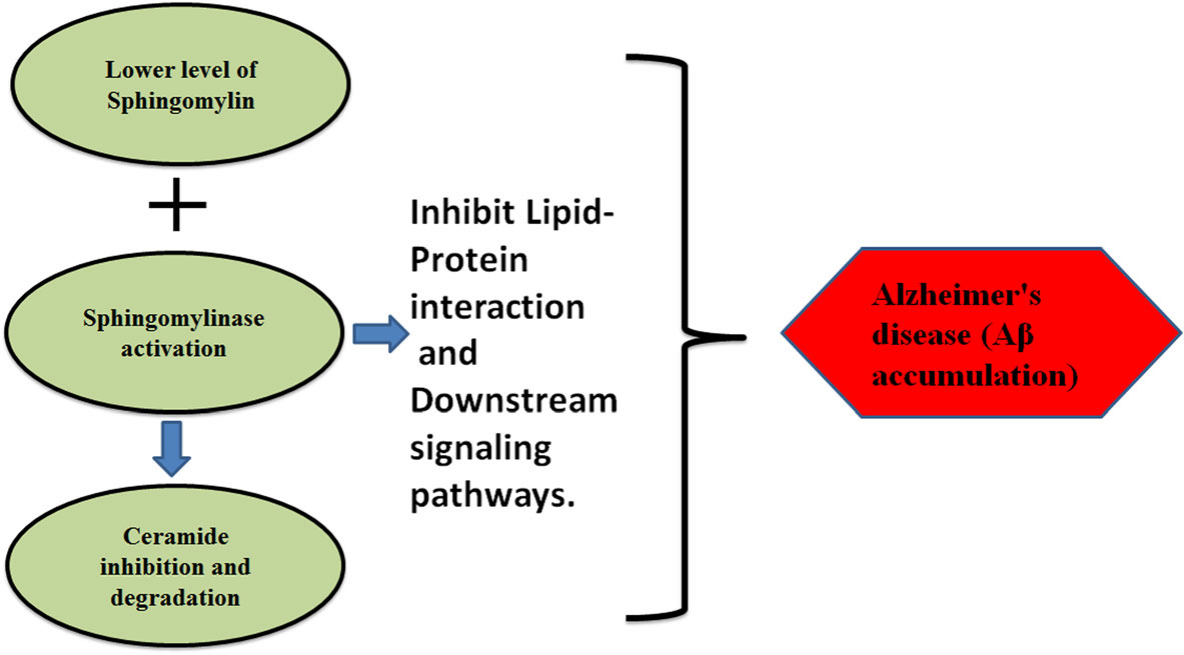
Sphingolipids and AD pathogenesis

Alzheimer’s disease (AD) is mainly characterized by the formation of plaques followed by the accumulation of Aβ peptides. Numerous key enzymes are also involved in the progression of AD including neprilysin, BACE-1 and a complex of γ-secretase play a critical role in the development and treatment of AD. Ceramide and SM are the two major classes of sphingolipids that can be transformed by sphingomyelinase (SMase) and SM synthase (SMSs) respectively. These are the important factors involved in the pathology of AD. Ceramides promote the generation and accumulation of Aβ by stabilizing the BACE-1 [96]. The SM binding motif is found in Aβ, that indicates that it is involved in the aggregation of Aβ [97]. The underlying mechanism may be the up-regulation of SM synthase-1 (SMS-1) which promotes the production of ceramide at the upregulated site of BACE-1. Recently, this idea was validated through experimentation. It was demonstrated that SMS-1 knockout in the hippocampus of AD mice lead to the attenuation of plaques of Aβ and neuroinflammation which reduced the cognitive declines. Moreover, SMS-1 knockout/inhibition also reduced the stability of BACE-1 by lysosomal deterioration of BACE-1 through controlling the intra-cellular BACE-1 trafficking [98]. Moreover, another enzyme known as glucosylceramide synthase (GCS) also worsens the symptoms of AD by catalyzing the biosynthesis of gangliosides, particularly ceramides. Recent work indicates that genetic deletion of GCS in the neurons of adult forebrain improves the spatial memory and reduces the damage of dendritic spines in the dentate gyrus of hippocampus [99]. Intriguingly, the Sphingosine-1-phosphate (S1P) influences the proliferation, cellular survival, cell proliferation, synaptic plasticity, and neurotransmitters secretion. The reduction of S1P receptor 1 (S1PR-1) is also involved in the pathology of AD. Moreover the S1P concomitant genes including ceramide synthases (CERS-1, CERS-2) and Sphingosine-1-phosphate lyase (SGPL-1) were found to be up-regulated in AD patients, whereas sphingosine kinases (SphK-1, SphK-2), ceramide kinase (CERK), and anti-apoptotic Bcl-2 were found to be reduced [100]. Hence, the spreading of the plaque formation is thought to be followed by the phosphorylated Tau and ceramide-enriched Aβ exosomes [101, 102]. It is important to note that by lowering exosomes ceramide, plaques and phosphorylated Tau, the progression of AD can be delayed. All these factors point towards the ceramide/SM balance and contribution of ceramide in AD development. Following picture describes the role of altered sphingolipids metabolism in AD pathogenesis Fig. 5.

### Sphingolipids in neurological and psychiatric disorders

The implications of altered sphingolipids metabolism in neurological and psychiatric disorders have been reviewed extensively. This impaired metabolism either involves the degradation pathways or biosynthesis of sphingolipids and their metabolites. Moreover, there is very limited data available on this subject.

#### Epilepsy

Epilepsy is a disorder with abnormal brain activity, causes unusual behavior and continuous episodes of seizures. Dysregulated metabolism of lipids is an important factor of modified activity of brain. Similarly, the altered metabolism of sphingolipids also points towards its crucial role in the pathogenesis of epilepsy. In this context, the possible mechanistic approach relies on the heterozygous deletion of CERS2 (Ceramide synthase 2) gene and the homozygous mutation in CERS1 and CERS1 gene is primarily involved in the synthesis of C18-ceramide. In neuroblastoma, CERS1 down-regulation initiates pro-apoptotic pathways and induces ER stress. The CERS-2 is known for maintaining membrane integrity and mutation in this gene leads to the detachment and degeneration of myelin sheath and ultimately inadequate neuronal myelination results in their deterioration [79]. Unfortunately, there is a dearth of data regarding the role of sphingolipids in epilepsy and more work is needed to decipher its role. Moreover, CERS1 deficiency also lowers the level of Myelin-associated glycoproteins (MAG) in oligodendrocytes, which indicates the impact of lipid composition of neuronal membranes on the expression of proteins [103, 104]. Moreover, the mutation in CERS-1 with increased production of C-18 ceramide has specifically been observed in progressive myoclonic epilepsy (PME) type-8 [105].

### Effect of dietary habits and nutraceuticals on Cholesterol and Sphingolipids metabolism

Dietary sources are accounted as major contributors of cholesterol and sphingolipids levels within the living system. Similarly, nutraceuticals, the dietary supplements are also taken for their potential advantageous health effects. Nutraceuticals provide health benefits and are related to functional food. Thus, they affect the lipid metabolism to a great extent by different biochemical pathways [106]. Adequate amounts of nutraceuticals and cholesterol is an important factor for the physiological functioning of the brain. So, the required quantity of cholesterol and sphingolipids can be derived from dietary sources and nutraceuticals. Consumption of high cholesterol foodstuff including egg, meat, butter, milk and many others result in higher cholesterol level within the body. In this manner, increased cholesterol utilization is compensated by bile excretion. At the same time, body diminishes the endogenous production of cholesterol to overcome high cholesterol [107]. Thus, in order to prevent the high cholesterol levels in plasma, a compensatory mechanism is helpful to regulate its physiological amount in the body. While pointing the role of nutraceuticals in the regulation of cholesterol metabolism, it is important to describe their positive association. They are said to exhibit lipids lowering effects and also have pharmacological applications. Most of this effect is derived by their cholesterol lowering ability and they are used against hypercholesterolemia [108, 109].

Sphingolipids and their metabolites are regulated by increased or decreased dietary intake. Although, all foods contain a good amount of sphingolipids but milk, eggs, fish, chicken liver and cottage cheese are especially rich in SP. In regard to the dietary habits, the western diets contain SM in concentrations of approximately 200–400 mg/day [110]. Milk and butter are accounted as major sources of sphingolipids, thus their higher intake results in up-regulated sphingolipids level. The higher sphingolipids level can affect the function of general health as well as brain health [111]. Although, a normal level is important for the synaptic functioning in the brain, but altered levels exhibit abnormal effects. The available data are insufficient to demonstrate the role of nutraceuticals in relation to the regulation of sphingolipids metabolism. Further research on these aspects is needed to explore the association between these factors.

## Conclusion

Cholesterols plays a key role in the maintenance of brain health. It serves as a fundamental constituent of myelination and is also essential for development and functional outcomes. Sufficient availability of cholesterol for synapse formation is a critical step in the structural and functional development of our nervous system. Following a nerve injury, cholesterol is required for axonal regeneration. Previous studies have proved that the injured nerves positively respond to the exogenously supplied cholesterol. Thus, targeting cholesterol metabolism may provide a way to combat problems with regeneration of injured nerves. Literature suggests that controlling the cholesterol transportation or improving the cholesterol availability in NDDs may provide protection or may delay the onset of disease. Being an important part of membrane lipid rafts, sphingolipids play a vital role in the life cycle of cells. There are different types of sphingolipids that exist in the brain and contribute structurally in membrane microdomains formation. Although, this review has illustrated various underlying mechanisms of cholesterol and sphingolipids metabolism in relation to the brain health and physiology, but further rigorous research is required for the evaluation of other antecedent facts. Moreover, in this review, the association between impaired cholesterol and sphingolipids metabolism and NDDs, neurological and neuropsychiatry diseases has also been discussed in detail. Further research is critical for the advancement of the therapeutical applications of cholesterol and sphingolipids metabolism targeting agents depending on their effects on impaired metabolism in different diseases.

## Abbreviations

AD: Alzheimer’s Disease
BDN: Brain-Derived Neurotrophic Factor
CERS: Ceramide synthase
CNS: Central nervous system
GlcCer: Glycolipid glucosylceramide
HD: Huntington’s Disease
LDLR: Low-density lipoproteins receptors
LDLs: Low density lipoproteins
MAG: Myelin-associated glycoproteins
NDDs: Neurodegenerative disorders
PD: Parkinson’s Disease
PNI: Peripheral nerve injury
RGCs: Retinal ganglion cells
SP: Sphingolipids
